# Is quantitative ultrasound a measure for metabolic bone disease in preterm-born infants? A prospective subcohort study

**DOI:** 10.1007/s00431-021-04081-4

**Published:** 2021-04-22

**Authors:** A. de Lange, J.M. Maaskant, M.M. van Weissenbruch

**Affiliations:** 1 Division of Neonatology, Department of Pediatrics, Amsterdam UMC, Location VU University Medical Center, De Boelelaan 1117, 1081 HV Amsterdam, The Netherlands; 2 Department of Clinical Epidemiology, Biostatistics and Bioinformatics, Amsterdam UMC, Location Academic Medical Center and University of Amsterdam, Amsterdam, The Netherlands

**Keywords:** Bone development, Metacarpal bone speed of sound, Metacarpal bone transmission time, Preterm infant

## Abstract

In this study, we aimed to (a) evaluate postnatal changes in bone development in relation to growth and (b) to determine factors associated with bone development, from birth to 24 months of corrected age. The metacarpal speed of sound (mcSOS) and metacarpal bone transmission time (mcBTT) were used to evaluate bone development in 98 preterm infants, during hospitalization and follow-up. The mcSOS and mcBTT values not only declined in the first 6 weeks of hospitalization but also during follow-up. The mcSOS reached its lowest point at 12 months (*β*=-34.64), while the mcBTT reached a plateau between 12 and 24 months (*β*=0.06). Univariable analysis showed that gender (*p*=0.28), time (*p*<0.001), and growth parameters (*p*<0.001) were significant negative associated factors with mcSOS, whereas with mcBTT, time (*p*=0.009), length (*p*=0.063), length standard deviation scores (SDS) (*p*=0.027), head circumference (*p*=0.005), and head circumference SDS (*p*=0.007) were significant positive. The multivariable model revealed that time (*β*= -3.364, *p*=<0.001), weight (*β*=-0.007, *p*<0.001) and length (*β*=1.163, *p*<0.001) for mcSOS and length (*β*=-0.021, *p*<0.001), and length SDS (*β*= 0.066, *p*<0.001) and head circumference (*β*=0.049, *p*<0.001) for mcBTT remained highly significant associated factors.

*Conclusion*: The most important finding is that mcSOS decreased and the mcBTT reached a plateau to 24 months. In both mcSOS and mcBTT, the growth parameters were significant factors.

*Clinical Trial Registration:* N/A

**Table:** 

**What is known:** • *Metabolic bone disease is one of the possible long term adverse outcomes after preterm birth.* • *Metacarpal speed of sound (mcSOS) and metacarpal bone transmission time (mcBTT) decline in the early postnatal period.*	
**What is new:** • *During follow-up, mcSOS further decreased and reached its lowest point at 12 months, while the mcBTT reached a plateau up to 24 months.* • *Postnatal nutrition in relation to comorbidity does not meet the optimal mineralization rate of the developing preterm bone.*

**Supplementary Information:**

The online version contains supplementary material available at 10.1007/s00431-021-04081-4.

## Introduction

Premature infants are at risk to develop metabolic bone disease (MBD), because they miss out the last phase of active bone mineralization in the womb and are therefore born with a significantly lower mineral bone store than term infants. The incidence of MBD is 55% of the infants < 1000 gram and 23% of the infants < 1500 gram at birth. With the emerge of MDB, two most common conditions of the premature skeleton play a role: osteopenia and osteomalacia [1, 2].

After birth, a sufficient nutrient intake of calcium, phosphate, and vitamin D is crucial for bone development [1, 3, 4]. Neonatologists are continuously optimizing the nutrition to the needs of preterm infants. However, it is still a challenge to meet the same mineralization rate inside the womb as outside the womb. Delayed enteral feeding or dependency on total parental nutrition (TPN) predisposes MBD [1, 5–7]. Studies showed that several risk factors are associated with the development of MBD, for example, treatment with loop diuretics and/or steroids affects bone mineralization [8–10]. Ali et al. reported a strong significant correlation between a higher dose of caffeine, administration for a longer period, and MBD [11]. In addition, infants who are more vulnerable for MBD have comorbidities, such as bronchopulmonary dysplasia (BPD), sepsis, or necrotizing enterocolitis (NEC). Gaio et al. showed that infants with BPD are more likely to develop MDB, due to lower energy intake, prolongation of the TPN period, and more days to regain birth weight, compared to infants without BPD [9]. In the case of NEC, bone resorption is increased and may be related to a long period of immobilization [12]. As a consequence of severe illnesses, infants need more TPN and mechanical ventilation days, which influence bone development [3–7, 9, 10, 12].

Several imaging technics are being used to screen for MBD: X-rays, dual-energy X-ray absorptiometry (DEXA), and quantitative utrasonography (QUS). Although DXA is regarded as a reference standard for assessing bone mineral status in adults and children, it has its limitations for the assessment of bone status in preterm infants: the need for neonatal transport to the scan, its sensitiveness for motion artifacts, and the exposure to cumulative dose of radiation. X-rays are not suitable for detecting mild MBD. Therefore, we have used the QUS to measure the metacarpal speed of sound (mcSOS) and the metacarpal bone transmission time (mcBTT). The clinical use of QUS is easy, has a short examination time, is radiation free, and is portable [13]. Up to date, most studies have measured the bone development from the admission of the infant to 36 weeks gestational age (GA). Only a few studies have reported repeated measurements of one or both parameters during a longer period [3, 14–17].

These longitudinal studies are different in duration of follow-up periods, which provides us limited data to evaluate changes in both SOS and BTT during and after the infants’ admission in the neonatal intensive care unit (NICU). It is essential to gain insight in changes in bone development and in factors that may influence MBD which can contribute to exceed strategies to prevent MBD.

In this study, we aim to (a) evaluate postnatal changes in bone development in relation to growth and (b) to determine factors associated with bone development, from birth to 24 months of corrected age.

## Methods

This project is reported according to applicable criteria of the Strengthening the Reporting of Observational Studies in Epidemiology (STROBE) Statement: guidelines for reporting observational studies.

### Study population

Our study incorporated a sample of 99 preterm infants, born between March 2009 and October 2014, who were admitted to our level 3 NICU of the Amsterdam UMC, Location VU University Medical Center (VUmc), the Netherlands. This is a substudy of a longitudinal observational study: “the Infant study”, that evaluated the immunogenetic, pharmacological, and neurodevelopmental aspects of nosocomial sepsis and meningitis in preterm infants born appropriate for gestational age (AGA) and preterm infants born too small for gestational age (SGA) [18]. Infants with a suspicion of nosocomial blood stream infection and/or meningitis (age at onset >72 hours) and with a GA<32 weeks or birth weight<1500 gram were included in the “Infant study.” Infants with (suspected) genetic or metabolic disorders were excluded. The researchers hypothesized that neuropsychological developmental outcome and growth and body composition in the first two years were negatively influenced by nosocomial sepsis and meningitis. Therefore, all infants were followed for growth, body composition which incorporated bone growth, and neurodevelopment outcome until they reached the corrected age of 24 months. The study protocol of the “Infant study” was approved by the Medical Ethics Review Committee of the VUmc, with protocol number 2008/77. Written informed consent was obtained from the parents. Previous studies revealed that certain factors influenced bone development [9, 10, 19]. As a pilot, we analyzed which factors were associated with MBD in infants with a clinical or proven sepsis. Due to a lack of information on the outcome parameter obtained from earlier studies in preterm infants, we did not perform a sample size calculation in our observational cohort study.

### Study measurements

The DBM Sonic Bone Profiler (IGEA, Carpi, Italy) was used to perform quantitative ultrasound (QUS) to evaluate bone development. The bone profiler uses ultrasound waves and has two 16 mm diameter probes which are placed at the opposite sides of the metacarpal bone and measures the mcSOS and mcBTT and are previously described in detail [15]. The emitting probe produces a frequency of 1.25 MHz and the device runs on Windows ME/XP software. According to the manufacturer, IGEA, the bone profiler has a sensitivity of 81.5 % and a specificity of 79.3%. The standardized reproducibility is 2.2% [20]. McSOS is expressed in meters per second (m/s) and mcBTT in microseconds [15, 20, 21]. BTT is independent of the amount of soft tissue around the bone [13, 21–23]. QUS gives information about the properties of the bone such as bone mineral density, cortical thickness, elasticity, and micro-architecture [13].

The ultrasounds were assessed from admission to discharge or death, and during follow-up at the corrected age of 0, 3, 6, 12, and 24 months, and were performed by trained research staff. One nurse was trained by the company, instructed other research staff, and supervised the first 10 measurements. During admission, QUS was performed weekly. Each session of QUS consisted of a series of four measurements with a tolerated range of 10 m/s. When measurement was out of range, the measurement was repeated, with a minimum measurement time of 5 and a maximum of 10 minutes.

Measurements were not performed if the infants were too unstable due to their clinical condition or if both hands were not available in case of cannula for intravenous infusions.

Neonatal data were collected from medical records. Baseline collection of patient characteristics: gestation in weeks, birthweight, length and head circumference, born AGA or SGA, gender, ethnicity (caucasian/non-caucasian or mix), and APGAR scores.

During admission and follow-up, we collected data on clinical characteristics (anthropometry, TPN days, mechanical ventilation days, and hospitalization days), medication (steroids, diuretics, antibiotics, and caffeine), comorbidities as sepsis, NEC (defined as Bell stage 2 or 3), BPD (defined as supplemental oxygen required at 36 weeks of gestation), intraventricular hemorrhage (grade 3 or 4), incidence of periventricular leucomalacia (PVL, grade 3 or 4), and mortality. During admission anthropometry measurements of length (cm), head circumference (cm), and naked bodyweight (gram) were assessed on a weekly basis and were measured to the nearest 0.1 cm, and body weight was measured to the nearest 5 grams. Preterm infants were classified as AGA if they had a birth weight that was above minus two standard deviation scores (SDS) or SGA if they had a birth weight below two SDS [24]. Infants were defined as very preterm if they were born between 28 and 32 weeks of gestation or extremely preterm if they were born before 28 weeks of gestation, based on the World Health Organization definitions of preterm birth [25]. Preterm infants were fed with a combined parenteral and oral nutrition directly after birth; the amount of fat, protein, glucose, electrolytes, and vitamins were administrated according to the standardized feeding Dutch guidelines [26]. Oral nutrition was preferably mother’s own milk, and when feeding reached 100 ml/kg/day, fortifier and 400-800 international units of vitamin D were added. Calcium and phosphate intake was prescribed as parenteral intake between 1.3-3 and 1-2.3 mmol/kg and enteral intake between 3-3.5 and 2.1-3 mmol/kg per day. If mother’s milk was not available, infants were fed by donor milk until 32 GA weeks or preterm formula.

During follow-up, length, head circumference, and naked weight were assessed at the expected date of delivery (40 weeks) and at the corrected age of 3, 6, 12, and 24 months. Length and head circumference were measured to the nearest 0.1 cm, and body weight was measured to the nearest 5 grams or 0.1 kg at 24 months.

### Statistical analysis

The data were analyzed using R statistics software, version 3.4.2. (R Foundation for Statistical Computing, Vienna, Austria). The statistical significance was defined at *p*<0.05, and statistically uncertainty was expressed by a 95% confidence interval (95% CI).

Continuous variables, if normally distributed, were reported as mean values and standard deviations (SD), otherwise, by median and interquartile ranges (IQR). Categorical variables were presented as number and percentage. Shapiro tests, histograms, plots, and Levene tests were performed to check normality and homoscedasticity.

Repeated measurements of mcSOS and mcBTT were analyzed with a multilevel model to reflect the pattern of change in bone mcSOS and mcBTT. This model is similar to standard regression models except that it accounts for possible clustering effects of repeated measurements over time. These clustered observations, made within one patient over time, maybe correlated, making a multilevel analysis necessary. We applied a two-level structure: repeated measurements of the patient are the lower level, while the patient is the higher level.

Besides growth parameters, we incorporated all earlier studied associated factors on bone development like treatment with a higher dose of caffeine [11], loop diuretics and/or steroids [8–10], delayed enteral feeding, dependency on TPN, or mechanical ventilation as consequences of severe comorbidities, [1, 3, 5–7, 9, 10, 12] in the univariable analyses.

Variables in the univariable analyses with a *p*<0.1 were included in the multivariable analyses. Variables with the highest *p* values were step by step, backwards removed from the analyses. Variables with a *p*<0.05 were considered significant and were obtained in the final analysis. To reflect the growth pattern, changes in weight, length, and head circumference and their respective SDS were also analyzed in a multilevel model.

## Results

### Baseline characteristics

In the initial cohort, 114 infants were included in the study. Of these infants, 15 infants were excluded from further analyses due to earlier drop out (13 infants died, 1 infant had an underlying medical condition, and parents of 1 infant withdrew consent).

Demographics, clinical characteristics of the remaining 99 infants are summarized in Table 1. Of these infants, 81.8% had proven sepsis and none of these subjects had a PVL grade 3 or 4.

**Table 1:** Demographics, clinical characteristics

Characteristic	Value
Demographics on birth	
Patients, *n*	99
Gestational age (weeks), mean (SD)	27.9 (2.0)
Male, *n* (%)	50 (50.5)
Birth weight (gram), median (IQR)	1045 (827.5-1257.5)
Birth weight (SDS), mean (SD)	-0.04 (1)
Birth length (cm), mean (SD)	36 (3.6)
Birth length (SDS), median (IQR)	0.04 (-0.85-0.6)
Birth head circumference (cm), mean (SD)	25.7 (2.5)
Birth head circumference (SDS), mean (SD)	0.09879 (1.1)
Extremely preterm, *n* (%)	46 (46.5)
Very preterm, *n* (%)	53 (53.5)
SGA, *n* (%)	22 (22.2)
Caucasian, *n* (%)	66 (66.8)
Non-Caucasian, *n* (%)	24 (24.2)
Mix (Caucasian/ non-Caucasian), *n* (%)	9 (9.1)
APGAR score after 5 min, median (IQR)	8 (7-9)
Clinical characteristics during NICU hospitalization	
Hospitalization NICU (days), median (IQR)	36 (22-51)
Total parenteral nutrition (days), median (IQR)	16 (12-22)
Endotracheal ventilation (days), median (IQR)	6 (2-14)
Nasal ventilation (days), mean (SD)	26.1 (13.9)
Oxygen (days), median (IQR)	13 (4-31.5)
Diuretics administrated (days), median (IQR)	1 (0-18.5)
Antibiotics administrated (days), median (IQR)	14 (9-23)
Caffeine administrated (days), median (IQR)	32 (18-48.5)
Proven sepsis (blood culture positive), *n* (%)	81 (81.8)
Treatment with glucocorticosteroids *n* (%)	13 (13.1)
NEC (>BEL2A,) *n* (%)	12 (12.1)
BPD (Oxygen at 28 days of life), *n* (%)	30 (30.3)
IVH (>grade 3, 4), *n* (%)	3 (3)

SGA: small for gestational age; NEC: necrotizing enterocolitis: BPD: bronchopulmonary dysplasia; IVH: intraventricular hemorrhage; SDS: standard deviation score; SD: standard deviation; IQR: interquartile range

### Postnatal changes in bone development

Ninety-eight subjects had at least two successful measurements of mcSOS and mcBTT during hospitalization and follow-up. QUS was performed during the first 6 weeks of admission until transfer to another hospital or death. In the first six weeks in hospital, a total of 558 scans were performed, respectively, 19, 41, 38, 35, 34, and 23 scans. During the five moments of follow-up, 81, 82, 81, 63, and 61 scans were made.

**Fig. 1:**
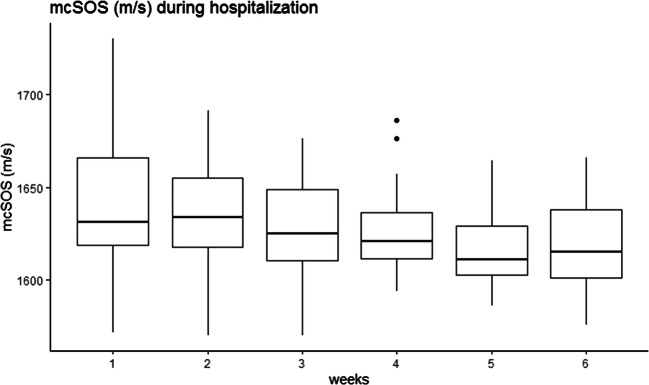
The mcSOS declined during the first five weeks of hospitalization. After five weeks, it seems to stabilize

**Fig. 2:**
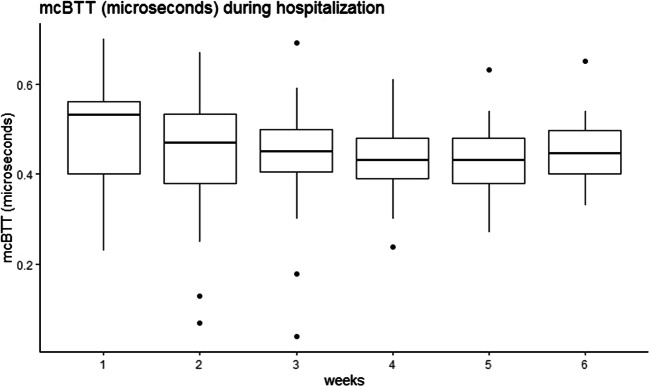
The mcBTT declined during the first four weeks of hospitalization. After four weeks, it seems to stabilize

**Fig. 3:**
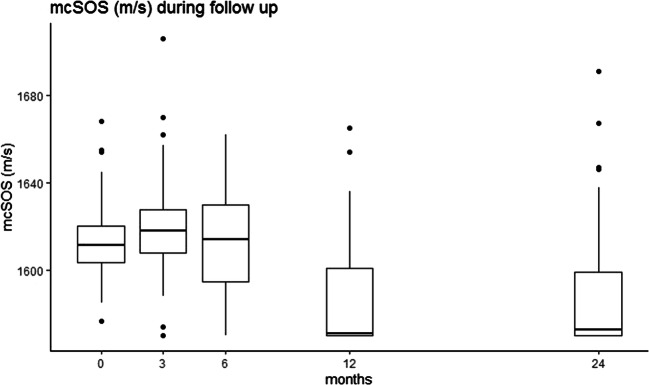
The mcSOS increased between 0 to 3 months; after this timepoint, the mcSOS declined and reached its lowest point at 12 months

**Fig. 4:**
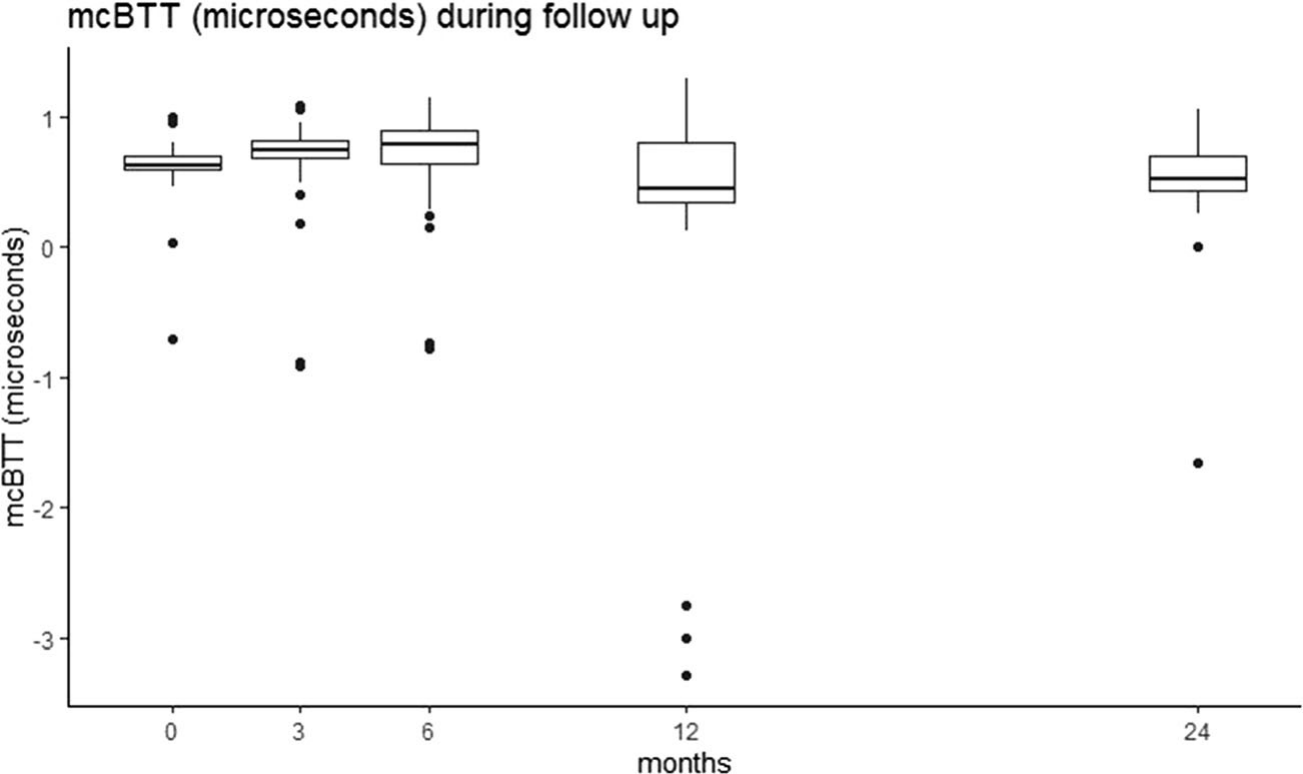
The mcBTT values increased between 0 to 6 months and decreased until 12 months. Between 12 and 24 months, a plateau was reached

The mcSOS and mcBTT values declined in the first 6 weeks of hospitalization and up to 24 months of corrected of age (Figs. 1, 2, 3, and 4). Time modeled as a categorical variable reflects the development of mcSOS and mcBTT over specific time points. There was a decrease of mcSOS, compared to the measurements during hospitalization that was taken as the reference. The lowest point in mcSOS was reached at 12 months of corrected gestational age (*β*=-34.64). In the mcBTT values, an increase was observed from 0 to 6 months. After 6 months, mcBTT decreased until 12 months. Between 12 and 24 months, a plateau of mcBTT (*β*=0.064) was reached. These results are summarized in Table 2.

**Table 2:** Changes for random effects of mcSOS and mcBTT over the time categories

Variables	Regression coefficient	Standard error	95% confidence interval		*p* value
			lower	upper	
mcSOS					
6 weeks of hospitalization	1622.10	2.77	1616.66	1627.53	<0.001*
0 months	-8.74	3.42	-15.45	-2.03	0.011*
3 months	-2.53	3.47	-9.35	4.29	0.466*
6 months	-11.42	3.55	-18.41	-4.44	0.001*
12 months	-34.64	3.88	-42.27	-27.01	<0.001*
24 months	-33.48	4.01	-41.37	-25.59	<0.001*
mcBTT					
6 weeks of hospitalization	0.45	0.01	0.40	0.49	<0.001*
0 months	0.18	0.02	0.11	0.28	<0.001*
3 months	0.26	0.04	0.19	0.37	<0.001*
6 months	0.24	0.04	0.17	0.35	<0.001*
12 months	-0.01	0.97	-0.14	0.07	0.307
24 months	0.06	0.05	-0.04	0.18	0.194

mcSOS: metacarpal speed of sound; mcBTT: metacarpal bone transmission time. *Significant *p* value <0.05

Post hoc analyses showed no significant difference in the (bone) growth pattern between subjects with a proven or non-proven sepsis: weight (*p*=0.124), length (*p*=0.118), head circumference (*p*=0.076), mcSOS (*p*=0.313), and mcBTT (*p*=0.685). Therefore, a subgroup analysis based on sepsis was not necessary.

### Factors associated with bone development

We performed univariable analyses to identify possible associated factors in bone development, measured by mcSOS and mcBTT. Gender, time, weight, weight SDS, length, and head circumference were significant negative associated factors; TPN days and NEC were significant positive associated factors with mcSOS. With mcBTT time, length, length SDS, head circumference, and head circumference SDS were significant positive associated factors. The results are summarized in Supplemental table S1 and S2 (online).

The significant variables from the univariable analyses were included in the multivariable models (Table 3). In mcSOS, time (*β*= -3.364, *p*=<0.001), gender (*β*=-5.296, *p*<0.037), TPN days (*β*=0.219, *p*<0.038), weight (*β*=-0.007, *p*<0.001), and length (*β*=1.163, *p*<0.001) remained significant associated factors. For mcBTT, length (*β*=-0.02, *p*<0.001), length SDS (*β*=0.066, *p*=<0.001), head circumference (*β*=0.049, *p*<0.001), and head circumference SDS (*β*=-0.054, *p*=0.016) were significant factors.

**Table 3:** Multivariable analysis for the fixed effects of mcSOS and mcBTT during hospitalization and follow-up

Variables	Regression coefficient	Standard error	95% confidence interval		*p* value
			lower	upper	
SOS					
Intercept	1601.77	11.07	1580.00	1624.53	0.000
Time	-3.36	0.97	-5.27	-1.46	<0.001*
Gender (male)	-5.30	2.50	-10.26	-0.34	0.037*
Total parenteral feeding days	0.22	0.10	0.01	0.43	0.038*
Weight	-0.01	0.00	-0.01	-0.00	<0.001*
Length	1.16	0.34	0.49	1.84	<0.001*
BTT					
Intercept	-0.02	0.14	-0.35	0.15	0.907
Length	-0.02	0.01	-0.04	-0.02	<0.001*
Length (SDS)	0.07	0.02	0.04	0.11	<0.001*
Head circumference	0.05	0.01	0.04	0.08	<0.001*
Head circumference (SDS)	-0.05	0.02	-0.11	-0.02	0.016*

mcSOS: metacarpal speed of sound; mcBTT: metacarpal bone transmission time; SDS: standard deviation scores; *significant *p* value <0.05

## Discussion

In this study, we aimed to (a) evaluate postnatal changes in bone development in relation to growth and (b) to determine factors associated with bone development, from birth to 24 months of corrected age.

We observed a decline in mcSOS in the first 6 weeks. Our finding was identical to other studies which also showed a decline in mcSOS [5, 14–17, 19]. A decline in SOS during the first weeks of life might be related to a postnatal inadequate mineral intake and illness. Surprisingly, during follow-up, mcSOS further declined and reached the lowest point at 12 months. This result was inconsistent with previous studies. Chen et al. reported a decrease of SOS in the first 4 months and then a step-by-step increase up to 12-15 months [14]. Furthermore, Tansug et al. found that SOS in preterm infants was decreased at 2 months but increased at 12 months [16]. To verify our mcSOS outcomes, we performed a post hoc analysis and modeled time as a categorical variable. This analysis confirmed our results. Other studies showed a decrease in SOS in preterm infants up to 6 months and then an increase to respectively 14 and 24 months [3, 15].

However, in term infants, lower SOS values were found at 12 months compared to values at birth [22]. Considering that term infants did not miss the last phase of active bone mineralization, the lower SOS may be explained by the dependency of soft tissue around the bone SOS [27]. Lower mcSOS values in our cohort during follow-up might be explained by multifactorial differences in nutritional status, supplementations, medications, physical activity, disease status, endocrine status, and exposure to environment in early life. Additionally, we are aware that family history of osteoporosis and genes predicts low bone mass among population [28].

In contrast to mcSOS, we found a different trend in mcBTT: a decrease during hospitalization, then an increase, and finally a plateau at 24 months. In agreement with other studies, mcBTT showed a similar decreasing pattern during the first weeks of life [7, 19]. In contrast, a different trend was reported by Ritschl et al., who demonstrated a continuous increase of mcBTT from birth up to 14 months in preterm infants [15]. In comparison to preterm infants, an increase in mcBTT from birth up to 12 months was found in term infants [22].

Differences in trends between mcSOS and mcBTT could be explained by the fact that SOS values are dependent of soft tissue thickness and subcutaneous fat, whereas BTT only measures bone properties [15, 27, 29]. In our cohort, we measured the metacarpal bone which is practically not influenced by soft tissue thickness. However, little is known about changes in BTT after discharge, since in most studies, only SOS was performed.

In our study, gender, time, and the growth parameters (weight, weight SDS, length, and head circumference) showed significant inverse associations with the decline of mcSOS. Only time, weight, and length remained highly significant after multivariable analysis. These results were consistent with Tansug et al. who reported a significant correlation between weight, length, and SOS, and Ritschl et al. who showed a negative correlation between weight, length, and SOS [15, 16]. Roggero et al. showed that GA and birth weight, but not birth length, were correlated with SOS [17], However, we did not find a significant association. In our study, both univariable and multivariable analysis showed that length, length SDS, head circumference, and head circumference SDS were significantly associated with mcBTT. Previous studies found a correlation between length and BTT [7, 15, 19]. Moreover, earlier was reported a significant correlation with both bone parameters and postnatal age [3, 4, 8, 14]. In our sample, postnatal growth in length and head circumference was correlated with mcBTT which we would have expected due to the maturity of the infant. Whether differences in gender play a role in bone development is not fully clarified because of contradictory findings in the literature. The negative association found in our study between the male gender and mcSOS might be in line with Zadik et al. demonstrating that bone mass during puberty in healthy girls increases more than in boys [30].

Furthermore, we have to take into account that acknowledged risk factors and preterm-related comorbidities negatively effects bone development. More days of mechanical ventilation and administration of postnatal steroids, diuretics, and TPN reduce bone mineral content and are inversely correlated to bone development [3, 5, 7, 10]. In contrast, we pooled the TPN days together with the days of TPN mixed with the administration of oral feeding which may have contributed to our positive association with mcSOS. Another important risk factor is NEC. Cakir et al. showed that NEC affects bone development due to increased bone resorption [12]. The incidence of NEC in our sample was 12.1%, and we assumed that it was too low to show an effect in the multivariable analysis.

This study has several limitations. First, QUS was used to evaluate bone development. Previous researchers used different devices and different skeletal sites which produced diverse bone reflection parameters. Second, the percentage of successfully performed scans dropped from 81.6% to 63.3%, due to lost to follow-up or measurement error. Measurement error was caused by non-cooperating behavior, when children reached the age of 12 and 24 months. These factors limit the generalizability of our results. On the other hand, mcSOS and mcBTT were measured both. To prevent measurement bias, the same research staff performed QUS. Third, we did not take postnatal mirco- and macronutrients in account. Since all infants were routinely checked on levels of phosphate, calcium, and alkaline phosphatase to prevent deficiencies. Finally, we did not include follow-up data such as nutritional status, supplementations, medications, physical activity and disease status, endocrine status, and exposure to the environment in our analyses. However, we realize that especially postnatal nutrition in relation to comorbidity does not meet the optimal mineralization rate of the developing preterm bone. It is important to explore if we could optimize bone development by making adaptions in feeding and/or supplementations with calcium and phosphate.

## Conclusion

Bone development measured by mcSOS and mcBTT decreased during hospitalization, mcSOS declined further, while the mcBTT reached a plateau up to 24 months. In both mcSOS and mcBTT, the growth parameters were significant factors. QUS could be a useful tool in a clinical setting to detect early signs of MBD. Future studies, including the accuracy and reproducibility of different devices and a longer follow-up, are necessary to evaluate changes in QUS parameters SOS and BTT; and to evaluate if factors as feeding characteristics, other environmental factors and genes influence bone development in the long term.

## Supplementary Information

ESM 1 (DOCX 18 kb)

ESM 2 (DOCX 29 kb)

## Abbreviations

AGA: Appropriate for gestational age
BPD: Bronchopulmonary dysplasia
CI: Confidence interval
DEXA: Dual energy X-ray absorptiometry
GA: Gestational age
IQR: Interquartile range
MBD: Metabolic bone disease
mcBTT: Metacarpal bone transmission time
mcSOS: Metacarpal speed of sound
NEC: Necrotizing enterocolitis
NICU: Neonatal intensive care unit
QUS: Quantitative ultrasound
SD: Standard deviation
SDS: Standard deviation scores
SGA: Small for gestational age
TPN: Total parenteral nutrition
