# Supplementary material for: Is quantitative ultrasound a measure for metabolic bone disease in preterm-born infants? A prospective subcohort study
Source: Eur J Pediatr. 2021 Apr 22;180(9):3009–17. doi: 10.1007/s00431-021-04081-4 (PMC8346443; doi:10.1007/s00431-021-04081-4)
Supplement: Supplementary file 1 — (DOCX 18 kb) [file 431_2021_4081_MOESM1_ESM.docx]

**TABLE S1:** Univariable analysis for mcSOS during hospitalization and follow-up

| **variables** | **regression coefficient** | **standard error** | **95% confidence interval**  **lower upper** | | **p-value** |
| --- | --- | --- | --- | --- | --- |
| **Demographics on birth** | | | | | |
| Gestational age (weeks) | -0.86 | 0.88 | -2.58 | 0.87 | 0.326 |
| Gender (male) | -7.46 | 3.34 | -14.08 | -0.84 | 0.028* |
| Birth weight | -0.00 | 0.01 | -0.02 | 0.01 | 0.479 |
| Birth weight (SDS) | 0.81 | 1.72 | -2.61 | 4.23 | 0.640 |
| Birth length | -0.29 | 0.50 | -1.28 | 0.70 | 0.573 |
| Birth length (SDS) | 0.62 | 1.47 | -2.29 | 3.53 | 0.674 |
| Birth head circumference | -1.12 | 0.70 | -2.52 | 0.28 | 0.116 |
| Birth head circumference (SDS) | -1.39 | 1.55 | -4.47 | 1.70 | 0.374 |
| Preterm class (extremely preterm) | 2.75 | 3.49 | -4.16 | 9.69 | 0.433 |
| SGA | -2.94 | 4.13 | -11.13 | 5.25 | 0.478 |
| **Clinical characteristics** | | | | | |
| Total parenteral nutrition (days) | 0.28 | 0.15 | 0.01 | 0.55 | 0.062* |
| Endotracheal ventilation (days) | 0.07 | 0.15 | -0.22 | 0.24 | 0.626 |
| Diuretics administrated (days) | -0.02 | 0.09 | -0.10 | 0.17 | 0.865 |
| Caffeine administrated (days) | 0.08 | 0.08 | -0.08 | 0.24 | 0.336 |
| NEC (yes) | 9.10 | 4.94 | -0.71 | 18.90 | 0.069* |
| BPD (yes) | 4.60 | 3.74 | -2.82 | 12.03 | 0.222 |
| **Growth during hospitalization and follow-up** | | | | | |
| Time | -2.68 | 0.30 | -3.28 | -2.09 | <0.001* |
| Weight | -0.00 | 0.00 | -0.00 | -0.00 | <0.001* |
| Weight (SDS) | -2.13 | 1.18 | -4.46 | 0.19 | 0.072* |
| Length | -0.73 | 0.08 | -0.87 | -0.58 | <0.001* |
| Length (SDS) | -0.73 | 0.96 | -3.38 | 0.25 | 0.445 |
| Head circumference | -1.52 | 0.16 | -1.84 | -1.20 | <0.001* |
| Head circumference (SDS) | -1.28 | 0.98 | -3.20 | 0.64 | 0.190 |

SGA: small for gestational age; NEC: necrotizing enterocolitis: BPD: bronchopulmonary dysplasia; SDS: standard deviation scores; SD: standard deviation; IQR: interquartile range.

mcSOS: metacarpal speed of sound; mcBTT: metacarpal bone transmission time

*significant p-value <0.1

**TABLE S2:** Univariable analysis for mcBTT during hospitalization and follow-up

| **variables** | **regression coefficient** | **standard error** | **95% confidence interval**  **lower upper** | | **p-value** |
| --- | --- | --- | --- | --- | --- |
| **Demographics on birth** | | | | | |
| Gestational age (weeks) | -0.01 | 0.01 | -0.02 | 0.12 | 0.556 |
| Gender (male) | 0.00 | 0.02 | -0.07 | 0.07 | 0.923 |
| Birth weight | 0.00 | 0.00 | -0.00 | 0.00 | 0.504 |
| Birth weight (SDS) | 0.02 | 0.02 | -0.01 | 0.06 | 0.238 |
| Birth length | 0.01 | 0.01 | -0.01 | 0.00 | 0.494 |
| Birth length (SDS) | 0.02 | 0.02 | -0.01 | 0.06 | 0.238 |
| Birth head circumference | 0.00 | 0.01 | -0.01 | 0.02 | 0.833 |
| Birth head circumference (SDS) | 0.02 | 0.02 | -0.01 | 0.05 | 0.254 |
| Preterm class (extremely preterm) | 0.01 | 0.03 | -0.06 | 0.06 | 0.929 |
| SGA | -0.03 | 0.04 | -0.11 | 0.05 | 0.516 |
| **Clinical characteristics** | | | | | |
| Total parenteral nutrition (days) | -0.00 | 0.00 | -0.06 | 0.00 | 0.454 |
| Endotracheal ventilation (days) | -0.00 | 0.00 | -0.00 | 0.00 | 0.938 |
| Diuretics administrated (days) | 0.00 | 0.00 | -0.00 | 0.00 | 0.781 |
| Caffeine administrated (days) | 0.00 | 0.00 | -0.00 | 0.00 | 0.773 |
| NEC (yes) | -0.00 | 0.05 | -0.11 | 0.10 | 0.963 |
| BPD (yes) | -0.02 | 0.03 | -0.10 | 0.05 | 0.546 |
| **Growth during hospitalization and follow-up** | | | | | |
| Time | 0.02 | 0.01 | 0.04 | 0.03 | 0.009* |
| Weight | 0.00 | 0.00 | -0.00 | 0.00 | 0.199 |
| Weight (SDS) | 0.00 | 0.02 | -0.03 | 0.03 | 0.844 |
| Length | 0.00 | 0.00 | -0.00 | 0.00 | 0.063* |
| Length (SDS) | 0.02 | 0.01 | 0.00 | 0.04 | 0.027* |
| Head circumference | 0.01 | 0.00 | 0.00 | 0.01 | 0.005* |
| Head circumference (SDS) | 0.03 | 0.01 | 0.01 | 0.06 | 0.007* |

SGA: small for gestational age; NEC: necrotizing enterocolitis: BPD: bronchopulmonary dysplasia; SDS: standard deviation scores; IQR: interquartile range.

mcSOS: metacarpal speed of sound; mcBTT: metacarpal bone transmission time

*significant p-value <0.1
